# Intensive Care Antifungal Stewardship Programme Based on T2Candida PCR and *Candida* Mannan Antigen: A Prospective Study

**DOI:** 10.3390/jof7121044

**Published:** 2021-12-06

**Authors:** Jannik Helweg-Larsen, Morten Steensen, Finn Møller Pedersen, Pia Bredahl Jensen, Michael Perch, Kirsten Møller, Birthe Riis Olesen, Mathias Søderlund, Maiken Cavling Arendrup

**Affiliations:** 1 Department of Infectious Diseases, Rigshospitalet, 2100 Copenhagen, Denmark; mathias.grauert.soederlund@regionh.dk; 2 Department of Intensive Care, Rigshospitalet, 2100 Copenhagen, Denmark; morten.steensen@regionh.dk; 3 Department of Thoracic Anesthesiology, Rigshospitalet, 2100 Copenhagen, Denmark; finn.moeller.pedersen@regionh.dk (F.M.P.); pia.bredahl.jensen@regionh.dk (P.B.J.); 4 Department of Cardiology, Section for Lung Transplantation, Rigshospitalet, 2100 Copenhagen, Denmark; michael.perch@regionh.dk; 5 Department of Neuro Anesthesiology, Rigshospitalet, 2100 Copenhagen, Denmark; kirsten.moeller.01@regionh.dk; 6 Department of Clinical Medicine, University of Copenhagen, 2100 Copenhagen, Denmark; maca@ssi.dk; 7 Department for Economy, Rigshospitalet, 2100 Copenhagen, Denmark; birthe.riis.olesen@regionh.dk; 8 Unit of Mycology, Statens Serum Institut, 2300 Copenhagen, Denmark; 9 Department of Clinical Microbiology, Rigshospitalet, 2100 Copenhagen, Denmark

**Keywords:** antifungal stewardship, invasive candidiasis, T2Candida, mannan antigen, diagnostic test

## Abstract

Non-culture-based biomarkers may improve diagnosis and antifungal treatment (AFT) of invasive candidiasis (IC). We evaluated an antifungal stewardship programme (AFSP) in a prospective intensive care unit (ICU) study, which included T2Candida and *Candida* mannan antigen (MAg) screening of patients with sepsis and a high risk of IC. Patients with non-neutropenic sepsis and a high risk of IC from two large tertiary ICUs were prospectively included, during a one-year period. IC was classified as proven, likely, possible or unlikely. The AFSP, diagnostic values of T2Candida and MAg, and the consumption of antifungals were evaluated. An amount of 219 patients with 504 T2Candida/MAg samples were included. IC was classified as proven in 29 (13.2%), likely in 7 (3.2%) and possible in 10 (5.5%) patients. Sensitivity/specificity/PPV/NPV values, comparing proven/likely versus unlikely IC, were 47%/100%/94%/90% for BC alone, 50%/97%/75%/90% for T2Candida alone, and 39%/96%/67%/88% for MAg alone. For the combination of T2Candida/MAg taken ≤3 days after AFT initiation, sensitivity/specificity/PPV/NPV was 70%/90%/63%/93%. T2Candida/MAg contributed to early (<3 days) AFT initiation in 13%, early AFT discontinuation in 25% and abstaining from AFT in 24% of patients. No reduction in overall use of AFT during the study period compared with the previous year was observed. An AFSP based on T2Candida and MAg screening contributed to a reduction of unnecessary treatment, but not overall AFT use. The diagnostic performance of T2Candida was lower than previously reported, but increased if T2Candida was combined with MAg.

## 1. Introduction

Invasive candidiasis (IC) comprises candidaemia and deep-seated tissue candidiasis [1,2]. The management of IC in intensive care units (ICU) is challenging. Definitive diagnosis of IC requires the detection of *Candida* from blood cultures (BC) or normally sterile tissues. However, the sensitivity of BC for diagnosis of IC ranges from 21% to 71% and is particularly low if few BC are taken, if antifungal therapy is already initiated and if multi-site infection is absent [3].

For high-risk ICU patients, randomized controlled trials of pre-emptive or empiric AFT have failed to demonstrate a survival benefit [4,5,6,7,8]. Untargeted AFT is common and may result in inappropriate treatment and emergence of drug resistance. Identifying at-risk patients is critical for rational use of AFT. However, although clinical risk predictors may help identify patients without risk of IC, they are not identifying ICU patients with high risk for IC satisfactorily [9,10].

The use of novel molecular and serological tests may contribute to the improved management of IC by earlier diagnosis and by detection of culture-negative IC. Currently approved non-culture-based assays include β-D-glucan, *Candida* mannan-antigen (MAg) and antibody (MAb) (MAg and Mab only in Europe), and the T2Candida magnetic resonance (T2MR) assay. The T2MR assay is able to detect *Candida* DNA in blood and was FDA approved in 2014, based on a study using laboratory spiked BC and BC from patients. That study reported a combined sensitivity/specificity of 91.1%/ 99.4% for the detection of candidaemia [11]. The following species can be detected by T2MR and are reported in three groups: (1) *C. albicans* and *C. tropicalis*; (2) *C. glabrata*, *C. krusei*, *Saccharomyces cerevisiae*, and *C. bracarensis*, which are reported together as *C. glabrata*/*C. krusei*; and (3), *C. parapsilosis*, *C. orthopsilosis*, and *C. metapsilosis*, which are reported as *C. parapsilosis*. These species account for approximately 95% of *Candida* blood stream infection (BSI) in Denmark [12]. Under optimal conditions, T2MR results can be available within 3–5 h, in contrast with BC, which may take several days to become positive [13]. Initial studies have shown high sensitivity in patients with candidaemia as well as in some patients with IC and negative BC during initial AFT [13,14].

For the detection of all different *Candida* species, *Candida* MAg and MAb, may be useful in patients at high risk of IC [14,15,16,17]. As the MAg test can detect infections with *Candida* species such as *C. dubliniensis*, *Clavispora lusitaniae*, and *C. kefyr* not included in T2MR that are potentially more common in ICU patients [18], we hypothesized that a combination of T2MR and Mag, in addition to the traditional methods, could improve the diagnosis and management of IC [19].

To our knowledge, the performance of combined T2MR and MAg in the setting of an antifungal stewardship programme (AFSP) has not yet been reported. We evaluated a new AFSP which included T2MR and *Candida* MAg screening in high-risk ICU patients.

## 2. Material and Methods

During 2017 and 2018, patients were enrolled from two large ICUs (the General and the Thoracic ICU, total bed capacity, 41 beds) at Rigshospitalet, University of Copenhagen, the largest Danish tertiary hospital with a total bed capacity of 1200 beds and a total ICU capacity of 62 beds.

Patients with non-neutropenic sepsis were eligible. Entry criteria included a minimum of 3 days of mechanical ventilation, and signs of persistent infection of uncertain cause despite antibacterial treatment, together with at least one of the following IC risk-factors: abdominal sepsis; renal replacement therapy; *Candida* colonisation ≥2 sites; total parenteral nutrition (TPN); or prednisolone >0.5 mg/kg for more than 7 days. *Candida* colonisation was defined as positive if yeast had been detected by microscopy or culture from microscopy, or culture from a non-sterile site within an interval of seven days before to three days after inclusion [20]. Patients with severe neutropenia and/or immunosuppression were excluded. Fluconazole prophylaxis was used for recent abdominal surgery with gastrointestinal perforation or anastomotic leakages or necrotizing pancreatitis.

**Figure 1:**
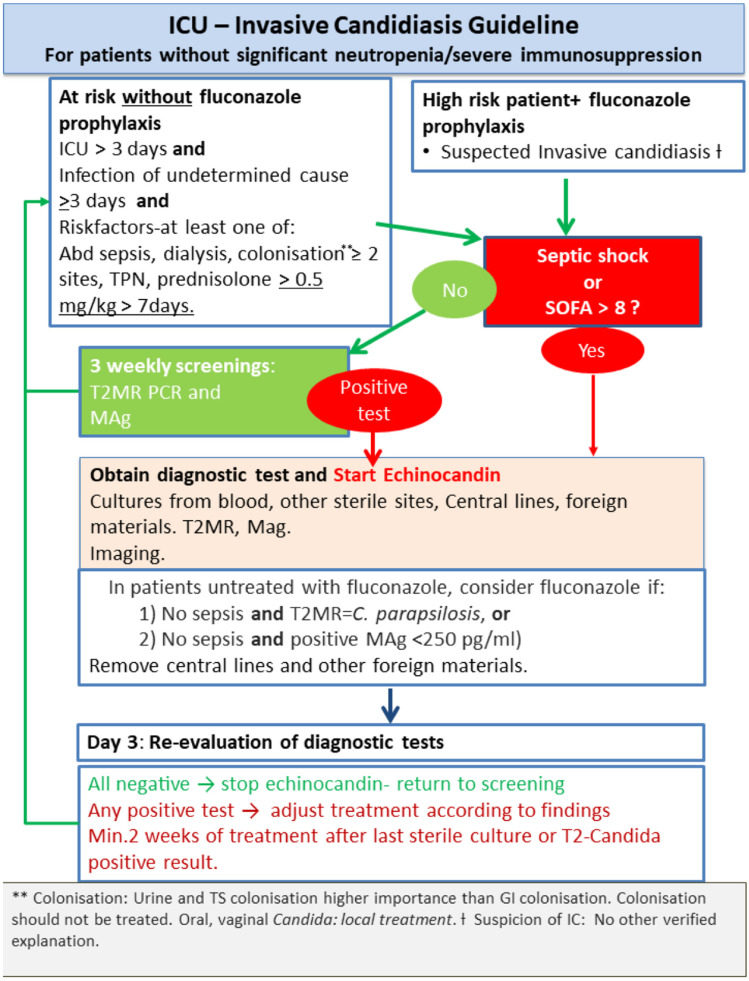
Antifungal Stewardship Programme flowchart algorithm.

The AFSP algorithm included diagnostic screening twice weekly, with T2MR (T2 Biosystems, Lexington, MA, USA) and Platelia *Candida* MAg (Bio-Rad, Marnes-la-Coquette, France) in accordance with the manufacturers’ instructions, in addition to traditional diagnostic tests until ICU discharge (Figure 1) and guided clinical evaluation, screening and AFT. The 1,3-beta-D-glucan test was not used.

IC was classified as proven, likely, possible, or unlikely, adopted from our previous publication [19]. *Proven IC*: (1) Growth of *Candida* in a BC, or (2) fulfilling all of: (a) growth of *Candida* in a tissue biopsy or sample from a drain placed within 24 h; (b) sampling from a normally sterile site using sterile procedures; (c) clinical or radiological signs of infection at that site (EORTC/MSG criteria [21]). *Likely IC:* No alternative microbiological diagnosis (+/−3 days) and severe sepsis despite 3 days of broad-spectrum antibacterial treatment with: (1) *Candida* isolated from at least two nonsterile sites (+/−3 days); and (2) *Candida* MAg >250 ng/L or T2MR positive. *Possible IC:* Sepsis and clinical illness compatible with IC despite 3 days of broad-spectrum antibacterial treatment and either: (1) *Candida* MAg >125 ng/L or T2MR positive and *Candida* isolated from at least 2 non-sterile sites (+/−3 days); or (2) colonised with the same *Candida* species from at least 2 non-sterile sites (+/−3 days); *No IC:* Remaining cases.

If IC was suspected or confirmed, central venous catheter (CVC) removal, echocardiography and ophthalmoscopy were strongly recommended. The AFSP was implemented as a non-compulsory stewardship programme through educational meetings, pocket leaflets and weekly consultations between specialists in intensive care medicine, infectious diseases and clinical microbiology. After a negative *Candida* MAg and T2MR, the treating ICU physicians were encouraged to discontinue AFT. Ultimately, the decision of treating or withholding AFT was the responsibility of the treating physicians.

The diagnostic value of T2MR and MAg in relation to classification of IC and traditional culture methods was evaluated. Overall consumption of antifungals in the two departments during the AFSP period and the preceding 2 years were compared. Clinical characteristics, test results, treatments, diagnosis of IC, and outcome were registered prospectively. The classification of IC was reviewed retrospectively, by three of the authors (JHL, MCA and MoS).

### Statistical Analysis

Categorical variables were compared by the chi-squared tests or Fisher exact tests, as appropriate. Continuous variables were compared using non-parametric Wilcoxon rank-sum tests. Risk factors and time to IC was investigated by log-rank models and Cox analysis. Analyses were done with Stata 15.1 (StataCorp).

## 3. Results

In total, 219 patients with 504 T2MR/MAg sample sets were included from September 2017 to October 2018. Most patients were male, severely ill with high rates of renal replacement therapy, total parenteral nutrition, acute surgery and vasopressor treatments (Table 1).

The number of test sets per patient was one in 41% (89/219), two in 30% (65/219), three in 11% (25/219) and ≥4 in 19% (41/219) of the patients. At the first test sets, 8% (18/219) of patients had received AFT for >3 days, whereas 130 of the 285 subsequent test sample sets (46%) were taken from patients who, at the time of sampling, had received AFT for more than three days. Overall, 13.7% (30/219) had positive T2MR; 11.0% (24/219) had positive MAg, and 8.2% (18/219) had positive blood cultures (BC). The T2MR test was invalid (negative internal control) in 8% (38/504) of samples.

IC was classified as proven in 13.2% (29/219), likely in 3.2% (7/219) and possible in 5.5% (12/219) of the patients (Table 2). The median time from admission to proven, likely or possible IC was 13 days (IQR 8–30). The incidence of IC was significantly higher in the General ICU than in the Thoracic ICU, OR 2.8 (95% CI: 1.2–6.9). The major underlying condition for proven and likely IC was abdominal emergency surgery (21/36, 59%) (Table 3). Intra-abdominal candidiasis was observed in 25 patients (11.4%) with 10 proven, 3 likely, and 5 possible cases. *C. albicans* and *C. glabrata* were the most common species in patients with proven and likely IC, followed by *C. tropicalis* and *C. dubliniensis* (Table 4). One positive *C. tropicalis* BC was regarded as a CVC contamination, as it was found three days after operation for a brain abscess, together with *S. epidermidis* in only a single of four BC with negative T2MR.

The overall 1-month (after study inclusion) and in-hospital mortality rates were 32% (71/219) and 41% (89/219), respectively. Patients with proven or likely IC had significantly increased overall mortality, compared with patients without IC, HR 4.4 (95% CI: 2.6–7.3) (Table 2).

### 3.1. Diagnostic Performance

**Figure 2:**
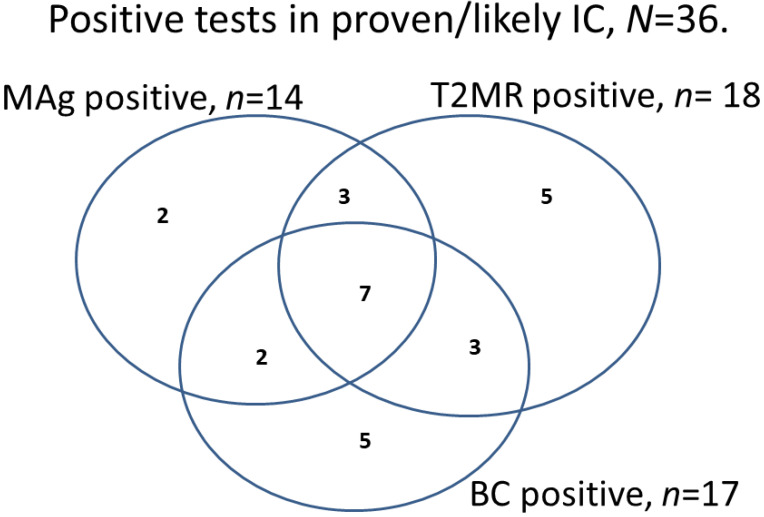
Positive diagnostic tests in patients with proven and likely invasive candidiasis. Abbreviations: BC, blood culture; MAg, mannan antigen; T2MR: T2Candida.

Details of T2MR and MAg findings in patients with candidemia and in patients with proven or likely IC without candidemia are shown in Table 5. A Venn diagram of positive test results for BC, T2MR and MAg in proven and likely IC is shown in Figure 2. Defining IC as proven/likely, compared with unlikely, sensitivity/specificity/PPV/NPV performance values for the entire patient population were 47%/100%/94%/91% for BC; 56%/97%/75%/90% for T2MR; and 39%/96%/67%/88% for MAg. MAg identified two patients with IC, in whom T2MR and BC were negative. Of these, one had proven IC with *C. lusitaniae*, which was not included in the T2MR panel. The best diagnostic performance was obtained with the combination of T2MR and MAg taken ≤3 days after AFT: sensitivity/specificity/PPV/NPV of 70%/90%/63%/93%, versus 61%/93%/63%/92% for all patients, respectively. Overall, similar diagnostic performance parameters were observed if IC was defined as proven/likely/possible (data not shown).

The combination of T2MR and MAg had sensitivity/specificity/PPV/NPV of 71%/79%/26%/96% for diagnosing candidaemia. In cases with BC-negative proven/likely intra-abdominal candidiasis (*n* = 13) specifically, sensitivity/specificity/PPV/NPV were 31%/89%/15%/95% for T2MR, 31%/92%/20%/96% for MAg and for combined T2MR and MAg, and 46%/84%/15%/96% if tests were taken ≤3 days after the initiation of AFT. Since several patients had possible abdominal IC, we also examined the diagnostic performance with the inclusion of possible BC-negative intra-abdominal candidiasis in the definition of true positives (*n* = 18). In this case, the sensitivity/specificity/PPV/NPV values for tests ≤3 days after AFT were 32%/90%/30%/91% for T2MR; 33%/93%/30%/94% for MAg, and 56%/86%/26%/96% for T2MR and MAg combined.

Discrepant T2MR/MAg results were observed in 55 of 504 (11%) test sets. On an individual patient basis, discrepant T2MR/MAg was observed in 12/36 (33%) cases, with proven or likely IC and in 11/171 (6%) of patients with unlikely IC.

### 3.2. Therapy

A total of 52 patients (24%) were managed without empiric or targeted AFT during hospitalisation (Table 6). The median cumulative duration of AFT, including both prophylactic, empiric and targeted treatments, was 10 days (IQR 5–19) for patients without IC, compared with a median of 18 days (IQR 12–38) in IC defined as proven, likely or possible.

A positive T2MR and/or MAg test contributed to initiation of early (<3 days) AFT in 13/101 patients (13%), of which nine had IC. Further, a negative T2MR and MAg test contributed to rapid (<4 days) discontinuation of AFT, or to patients not receiving AFT at all in 24% (12/50).

Systemic administration of AFT (defined as amphotericin B [ambisome], echinocandins, voriconazole, posaconazole, isavuconazole, or fluconazole at a minimum dosage of 400 mg) was analysed for all patients treated both within and outside of the study during the study period, compared with the previous year. The cumulative number of hospitalisation days were comparable; General ICU/Thoracic ICU resulted in 15,324/9604 days during the study period, and 14,557/8454 days the preceding year. When comparing the study period with the preceding year, there were no significant differences in the overall median or mean duration of systemic AFT; thus, in the General ICU, median/mean echinocandin treatment days were 6/11 versus 6/15 days per patient treated with an echinocandin, and mean echinocandin daily defined doses (DDD) were 71 versus 74 per month. In the Thoracic ICU, median/mean echinocandin treatment days were 10/24 versus 10/13 days, and mean overall echinocandin DDD per month was 38 versus 32.

## 4. Discussion

Recent guidelines recommend non-culture based diagnostic testing in addition to blood culturing in centres with frequent invasive fungal diseases, but without specific recommendations as to which tests to use and how [22,23,24,25]. The combined use of T2MR and MAg has, to our knowledge, not previously been examined for antifungal stewardship in clinical practice. We present our results from a one-year prospective AFSP study of high-risk ICU patients, who had a high rate of proven and likely IC (16.4%). T2MR and MAg contributed to appropriate therapy but were not associated with a change in the overall use of echinocandin or azole treatments, compared with the previous year in contrast to the findings of others [25]. An improved diagnostic performance was confirmed when T2MR and MAg were combined, in agreement with previous observations [19]. For diagnosis of abdominal IC, the sensitivity of the T2MR test alone was relatively modest (32%) and similar to BC (30%). However, an improved sensitivity of 45% for diagnosis of IAC was observed for the combination of T2MR and MAg. Altogether, the diagnostic performance of T2MR for diagnosis of IC was higher than for BC and MAg individually, but considerably lower than initially reported for the detection of candidaemia by T2MR [11,14].

### 4.1. T2MR

The use of T2MR for diagnosis of candidemia and deep-seated IC has recently been reviewed [26]. In a meta-analysis of eight studies by Tang et al., a pooled sensitivity of 91% (95% CI: 0.88–0.94) and specificity of 94% (95% CI: 0.93–0.95) for the diagnosis of candidemia was found. Several observational studies have suggested that the use of T2MR in conjunction with blood cultures could enhance antifungal stewardship in patient populations at risk of invasive candidiasis [27,28,29,30]. Gill et al., reported that the use of T2MR was associated with a significant decrease in the duration of empiric echinocandin use in a setting with a low prevalence of candidaemia (2% in the T2MR arm) [29]. Patch et al. found improved time to initiation of antifungal therapy, and avoidance of empiric antifungal treatment by comparison of a pre and post-implementation T2MR period, in which 6% (20/325 patients) had a positive T2MR [28]. In two Spanish studies (*n* = 49 and *n* = 30) T2MR was more efficient in predicting complicated candidaemia and the outcomes of antifungal therapy for suspected candidiasis, compared with cultures [31,32].

In agreement with our study, several other recent studies have reported lower diagnostic performance of the T2MR assay, compared with initial studies. Bomkamp et al., reported a sensitivity/specificity/PPV/NPV of 65%/96%/41%/99% for the detection of candidemia at a 4.4% pretest likelihood of candidemia [30]. Arendrup et al. [19]. Reported a sensitivity/specificity/PPV/NPV of 59%/96%/83%/94% for diagnosing candidaemia and deep-seated IC. Probably, the discrepancy between the initially reported T2MR performances and more recent findings relates to a higher inclusion of blood culture negative IC [11,14,16]. Compared with most previous studies, our patients had a higher severity of disease, higher rates of prior antifungal prophylaxis, and a higher rate of blood culture negative IC [11,28,30].

In addition to the fact that T2MR does not detect *C. dubliniensis* and other less common and rare candida species, a potential limitation when using T2MR for the detection of invasive candidiasis is that the system does not discriminate between the sibling species within the *C. glabrata* and *C. parapsilosis* complexes.

### 4.2. Mannan Antigen

Mannan-Ag for diagnosis of IC has been examined in several mostly observational studies. In two systematic reviews of studies before 2014, pooled sensitivity and specificity of MAg ranged from 58–62% and 86–93%, respectively [33,34]. Subsequent studies have reported considerable variation in the diagnostic performance of MAg, with sensitivity ranging from 55 to 88%, and specificity ranging from as low as 15% up to 100% [35,36,37]. In a recent retrospective study of the EMPERICUS randomized clinical performance of BDG, the mannan-Ag, and mannan antibody were assessed in a mixed medical/surgical ICU population with an IC incidence of 11.5%. In this study, in which a majority of patients were non-surgical, MAg had relatively good NPV but poor specificity for prediction of IC with a sensitivity/specificity/PPV/NPV of 88%/15%/14%/89% [6,38].

### 4.3. Combination Biomarkers to Guide AFT

Only a single prospective study has previously assessed the use of combination biomarkers for the early discontinuation of AFT [39]. In an RCT of a biomarker strategy versus standard care to discontinue early empirical AFT (N = 109, subsequent rate of proven and probably IC only 5%), Rouze et al. reported that an algorithm based on β-D-glucan, MAg and mannan antibody was safe for the early discontinuation of empirical AFT in patients with suspected invasive *Candida* infections. That study did not assess the use of T2MR [39].

Deep-seated invasive abdominal candidiasis remains difficult to diagnose. In our study, the advantage of using combined T2MR and MAg testing, was most pronounced in invasive abdominal candidiasis (IAC). We observed that the sensitivity of the T2MR test alone for diagnosis of abdominal IC was relatively modest (32%), and similar to BC (30%). These findings agreed with two recent studies reporting sensitivity/specificity rates of 45%/96% and 33%/93%, respectively, for proven or likely deep-seated invasive candidiasis and IAC, respectively [19,40].

In comparison, we found an improved sensitivity of 45% for the diagnosis of IAC by use of combined T2MR and MAg, despite including patients on AFT.

### 4.4. Antifungal Use

Introduction of an AFSP programme may increase awareness of fungal infections that may alter prescription practices. Implementation of systematic and more frequent diagnostic testing could increase the use of early pre-emptive echinocandin AFT. Potentially, unnecessary antifungal treatment may increase due to a higher awareness of IFI (outside the defined study patients or until a negative result was available) or as a result of false positive test results. A precise cost calculation would have required a randomised or matched case control study, which was not feasible. However, the goal of AFSP is to increase the number of patients who receive early and appropriate therapy, not to reduce the overall cost and use of AFT. Therefore, it is comforting that the AFSP contributed to appropriate therapy without an increased use of AF. It is plausible that a setting with more frequent T2MR and MAg testing with strict adherence to the AFSP may even reduce the AF use through the earlier discontinuation of unnecessary therapy.

The tests are expensive, raising the question of cost efficiency in AFSP. Results must be interpreted according to the clinical probability of IC. Based on the number of IC (proven, likely and possible) screened at less than 3 days of AFT, the positive and negative diagnostic likelihood ratios for combined T2MR and MAg was 8.55 (95%CI: 4.6–15.85) and 0.26 (95%CI: 0.15–0.46). Assuming a 12% pre-test probability of IC, as observed in the thoracic ICU, the post-test probability of IC with negative T2MR/ MAg decreased from 12% to 3%, which is a reasonable level for withholding AFT. In comparison, in a high prevalence setting with an overall pre-test probability of 28% for proven/likely/possible IC, such as that observed in the General ICU, the posterior probability for IC is 10%. In such cases, a decision to withhold AFT needs to be evaluated carefully, particularly in patients with possible deep abdominal candidiasis, for which no test performed optimally.

Our study has limitations. We did not assess the use of the pan-fungal β-D-glucan marker, because our AFSP targeted the detection of IC and not invasive fungal infection in general, and because of the relatively long turnaround time of the β-D-glucan test, which required batching of samples. Further, the mannan antibody test was not assessed, due to the poor performance of this biomarker [19,37,41]. The rate of invalid T2MR test results was 8% of samples, but comparable with other studies reporting a 6 to 14% rate [11,16,42,43]. The AFSP algorithm demanded a minimum of a 3-day stay in the ICU with persisting sepsis before the first diagnostic screening (a few patients were also transferred from other hospitals), and therefore several patients were already receiving prophylactic or empiric AFT at inclusion. In cases with discrepant T2MR and MAg results, most patients were treated in accordance with the high-risk setting and IC prevalence of our population. Several patients experienced repeated episodes of suspected IC during prolonged hospitalisations. For many of these patients with intermittent sepsis of unknown origin, a return to bi-weekly screening proved unnecessary and a significant proportion of the patients were only tested once or twice. Finally, the comparison of antifungal consumption in the two time periods may be biased by variation in case mix, AFT prescription for other fungal diseases than IC, turnover of ICU practitioners with different prescription practices, and overtreatment resulting from positive results.

## 5. Conclusions

Our study suggested that the combination of T2MR and MAg had improved diagnostic performance, compared with T2MR alone, with a sensitivity of 70% and a high NPV of 93% in patients receiving ≤3 days AFT. T2MR and MAg as adjunctive non-culture-based diagnostic tools should be restricted to patients at significant risk of IC, and results need to be carefully interpreted according to the pre-test probability of IC. An AFSP with these tests may help to target AFT and withhold unnecessary AFT. This combination may contribute to improved management of IC, particularly if initiated before AFT prescription and with strict adherence to the AFSP.

## Figures and Tables

**Table 1:** Baseline characteristics at inclusion for the 219 included patients.

Baseline Characteristics, N (%)	Years/Days (IQR) or N (%)
**Demography and underlying diseases**	
Age, median years	63 (IQR 52–72)
Male sex	152 (69%)
Previous SOT or BMT	18 (8%)
Malignancy	50 (23%)
IDDM	15 (7%)
**Type of ICU, ventilation and pressor support**	
General ICU	141 (64%)
Thoracic ICU	78 (36%)
Days of mechanical ventilation, median (IQR)	11 (6–15)
Vasopressors at inclusion	102 (61%)
**Surgery**	
Surgery during hospital stay (%)	173 (79%)
Emergency gastrointestinal/hepatobiliary surgery and/or gastrointestinal perforation	46 (21%)
Thoracic surgery	78 (36%)
**Other risk factors**	
Renal-replacement therapy	77 (45%)
Total parenteral nutrition	63 (29%)
Chemotherapy ≤ 3 months	18 (8%)
Corticosteroid > 25 mg daily	27 (12%)
Other immunosuppressive treatment	20 (9%)
Blood transfusion receipt	157 (72%)
***Candida*** colonisation	186 (85%)
***Candida*** colonisation index ≥ 0.5 *	114 (52%)

*: More than half of the samples with positive colonisation *Candida* cultures (20); ICU: intensive care unit; SOT: solid-organ transplantation; BMT: bone-marrow transplantation.

**Table 2:** Departments, classification of IC cases, length of treatment and outcome.

Invasive Candidiasis (IC)	n/Totals (%)
General ICU, n. proven/likely/possible cases	39/141 (28%)
Thoracic ICU, n. proven/likely/possible cases	9/78 (12%)
**IC classification**	
Proven	29/219 (13.2%)
Likely	7/219 (3.2%)
Possible	12/219 (5.5%)
Unlikely	171/219 (78%)
**Clinical outcome**	
In hospital mortality	89/219 (41%)
Mortality in patients with proven/likely IC	21/36 (58%)
Mortality in patients without proven/likely IC	65/171 (38%)
Early Death (<5 days) with proven/likely IC	5/36 (13.9%)
Length of AFT ^a^, proven/likely IC, median days (IQR)	18 (11–38)
Length of AFT, no IC, median days (IQR)	10 (5–19)

^a^ AFT: Antifungal therapy.

**Table 3:** Overview of the major sites of infection and the proportion of patients with candidaemia or deep-seated infection only are indicated.

Sites of Infection	Total IC (*n* = 48)	Proven IC (*n* = 29)	Likely IC (*n* = 7)	Possible IC (*n* = 12)
Blood (candidaemia) ^a^	17 (35%)	17 (59%)	0	0
Blood stream only	6	6	0	0
Abdominal ^a^	25 (52%)	16 (55%)	4	5 (42%)
Deep-seated only	18	10	3	5
Thoracic/cardiac ^a^	10 (21%)	6 (21%)	3	1 (8%)
Deep-seated only	7	3	3	1
Burns ^a^	2 (4%)	1 (3%)	0	1 (8%)
Burns only	1	0	0	1
Other and/or multiple foci	5 (10%)	0	0	5 (42%)
Deep-seated only	5	0	0	5

^a^ Includes patients with both candidaemia and abdominal, thoracic/cardiac and burns wound infection.

**Table 4:** Causative *Candida* species based on positive cultures for patients with proven or likely invasive candidiasis.

Species	N of Patients with	
	Candidaemia	Proven/Likely IC, without Candidemia *
* C. albicans*	9	11
* C. tropicalis*	1	2
* C. glabrata*	1	3
* C. krusei*	1	1
* C. parapsilosis*	1	0
* C. dubliniensis*	3	0
Other *Candida* species	1	2
Total number of culture positive	17	19

* One positive BC with *C. tropicalis* was regarded as a contamination.

**Table 5:** Summary of positive T2MR and Mannan antigen test in patients with candidemia and in patients with proven or likely IC without candidemia.

Diagnostic Test and Results	Patients with Positive Tests, N	Candidaemia *N* of Positive Patients	Proven/Likely IC, No Candidemia *N* of Positive Patients
T2MR *Candida* species			
* C. albicans/C. tropicalis*	22	8/10	6/8
* C. glabrata/C. krusei*	6	1/2	2/2
* C. parapsilosis*	2	1/1	0/0
Mannan antigen positive			
≥125 ng/L	**24**	**9/17**	**5/10**

**Table 6:** Antifungal therapy, indications and drug classes among the 219 patients.

Antifungal Prescriptions	N (% of All)
No antifungal treatment	52 (24%)
Clinical indication for initial therapy	
Invasive candidiasis	26 (12%)
Other fungal infection	5 (2%)
Empiric anti-fungal treatment initiated	96 (44%)
Prophylactic antifungal treatment initiated	40 (18%)
Antifungal agents	
Fluconazole only	51 (23%)
Echinocandin only	48 (22%)
Mould-active azole or Ambisome *	26 (12%)
≥2 different antifungals given during admission	68 (31%)
De-escalation from echinocandin to fluconazole	34 (16%)

* At any time during ICU admission received voriconazole, posaconazole, isavuconazole or ambisome.

## Data Availability

Not applicable.
